# Prediction of long-term visual outcome of idiopathic full-thickness macular hole surgery using optical coherence tomography parameters that estimate potential preoperative photoreceptor damage

**DOI:** 10.1007/s00417-024-06500-2

**Published:** 2024-05-08

**Authors:** Beáta Bajdik, Attila Vajas, Gréta Kemenes, Mariann Fodor, Éva Surányi, Lili Takács

**Affiliations:** Department of Ophthalmology, Faculty of Medicine, University of Debrecen, Nagyerdei Krt 98, 4032 Debrecen, Hungary

**Keywords:** Idiopathic full thickness macular hole, Vision prediction, Optical coherence tomography

## Abstract

**Purpose:**

To identify optical coherence tomography (OCT) parameters that predict postoperative best corrected visual acuity (BCVA) and are based on recent understanding of the pathomechanism of idiopathic full thickness macular hole (iFTMH) formation and closure.

**Methods:**

A retrospective consecutive case series of patients who had macular hole (MH) surgery at our institution between 2016 and 2022 was performed. 32 eyes of 30 patients were selected with at least 12 months of follow-up, closed MH and good quality OCT at each visit. Univariate correlation analysis, multiple logistic regression with forward stepwise selection, and Akaike’s Information Criterion (AIC) were used to identify the best predictors for postoperative BCVA at 6 and 12 months (M), and final (≥ 12 M) visits, and a new OCT index was created. Abilities of best models/indices to predict < 0.30 logMAR (> 20/40) BCVA were compared to macular hole index (MHI) using the area under the receiver operating curve (AU-ROC) analysis.

**Results:**

Statistical analysis revealed base diameter (B) (6 M), preoperative BCVA and B (12 M) and smaller ELM-GCL distance (A), and B (final visit) as predictors for postoperative BCVA. AU-ROC analysis indicated greatest AUC at 6 M for MHI and B (0.797, *p* = 0.004 and 0.836 *p* = 0.001, respectively) and for the new A/B index at 12 M and final visit (0.844, p = 0.002 and 0.913, p = 0.003, respectively).

**Conclusion:**

Our study suggests that MHI and B can be useful predictors of short term BCVA while the new A/B index that incorporates OCT parameters indicating potential preoperative photoreceptor damage may be a good predictor for long term postoperative BCVA. Our findings support the theory that initial hole formation mechanisms and photoreceptor damage define visual prognosis.

**Supplementary Information:**

The online version contains supplementary material available at 10.1007/s00417-024-06500-2.


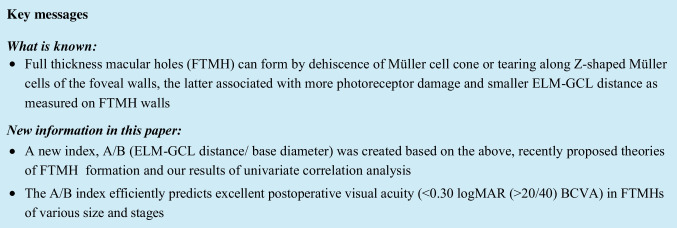


## Introduction

**Fig. 1:**
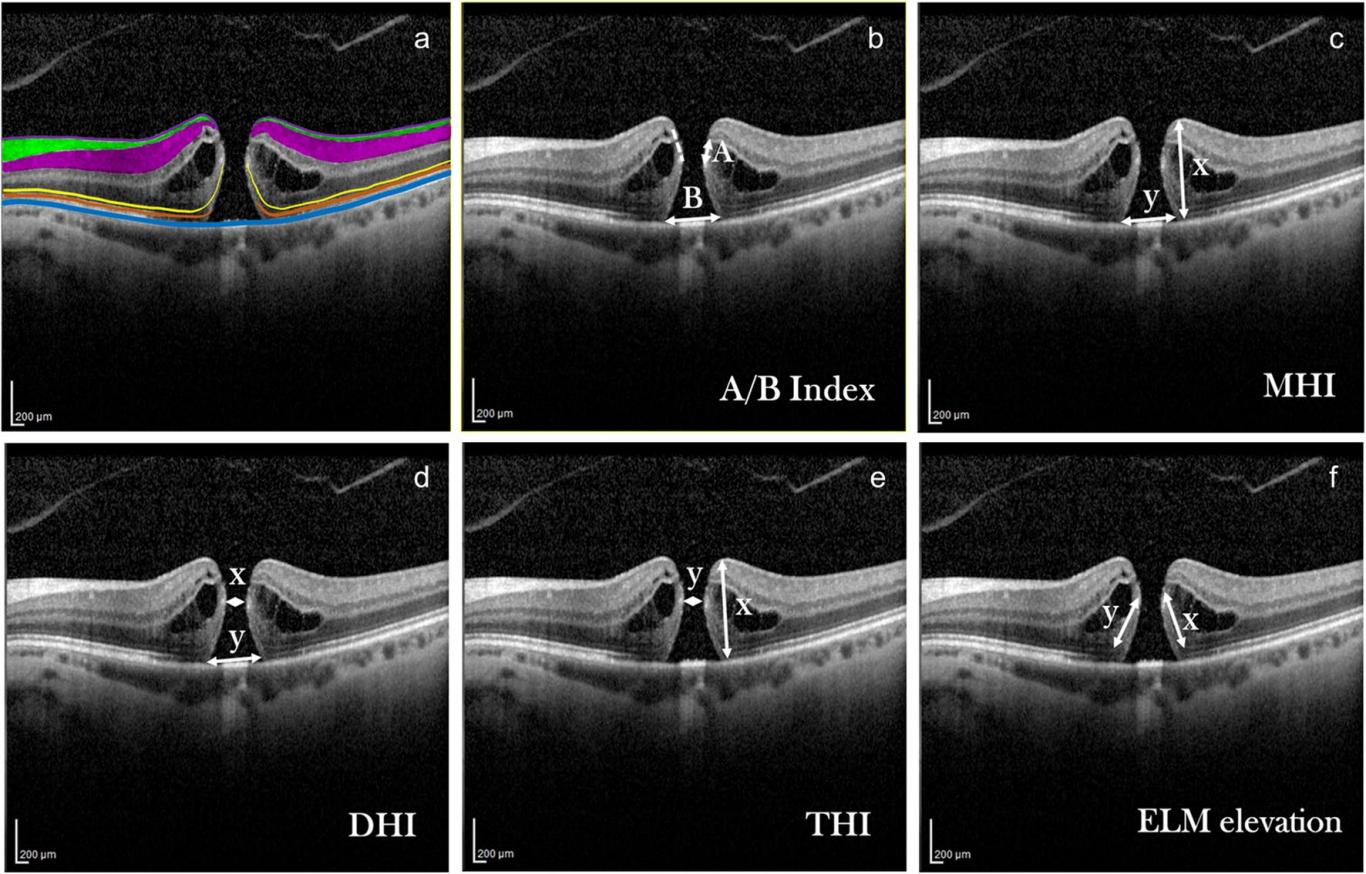
**a** Color coded annotation of the OCT layers. Violet-Internal Limiting Membrane; Green-Retinal Nerve Fiber Layer; Purple-Ganglion Cell Layer; Yellow-External Limiting Membrane; Orange-Photoreceptor Inner/Outer Segments; Blue-Retinal Pigment Epithelium. **b-f** Schematic presentation of the measured preoperative OCT parameters. All measurements were done at the largest macular hole (MH) diameter. A/B Index: smaller ELM-GCL distance(A)/base diameter(B) ELM-GCL distance was measured on both walls and the smaller was selected for calculation of the index; Macular Hole Index (MHI): macular hole height(x)/base diameter(y); Diameter Hole Index (DHI): minimum inner hole diameter(x)/base diameter(y); Tractional Hole Index (THI): macular hole height(x)/ minimum inner hole diameter(y); ELM elevation: the sum of ELM elevations on the nasal(y), and temporal(x) MH walls

Idiopathic full thickness macular holes (iFTMHs) cause important visual loss. Surgical treatment with pars plana vitrectomy has been first reported in 1991 [1] and since then, widespread use of vitrectomy and internal limiting membrane (ILM) peeling [2] as well as the introduction of inverted ILM flap technique [3] has resulted in closure rates of approximately 95% in several recent studies [4, 5] including over 90% success rate in the case of large holes (≥ 500 µm) [4]. However, in spite of successful surgical closure, postoperative VA is variable and excellent visual acuity (VA < 0.3logMAR or > 20/40) can be achieved in only ~50% of the cases [5, 6]. Many studies attempted to identify preoperative factors that could predict postoperative visual acuity. With the advent of SD-OCT technology, detailed analysis and measurement of foveal layers has become possible and OCT parameters are frequently used to predict surgical outcomes. Preoperative visual acuity, ELM defect, minimum hole diameter, duration of symptoms, and base diameter were shown to correlate with postoperative visual acuity [5, 7, 8]. Indices derived from several OCT parameters, such as macular hole index (MHI, maximum height/base diameter), diameter hole index (DHI, minimum hole diameter/maximum hole diameter) and tractional hole index (THI, maximum height/minimum hole diameter), were also introduced (Fig. 1), from which MHI proved to be useful in several studies and has become the most widely accepted [7, 9–11].

Earlier electron- and immunofluorescence microscopic examination of iFTMH opercula showed that 33% of opercula contained only glial elements, while 67% of the opercula contained one or more cone photoreceptor (PR) fragments and 50% of the opercula contained more than 5 PRs with cone PR somata involving cell nuclei and internal photoreceptor fibres, but lacking outer segments [12, 13]. Variable PR loss can be related to the existence of a specific population of Müller cells, the Müller cell cone (MCC) in the foveolar area, first described by Yamada in 1969 [14]. Gass proposed that the rupture of Müller cell cone (MCC) can occur without important damage to the foveal photoreceptor cells during macular hole formation since it was suggested to play a role only in the mechanical stabilization of the fovea [15]. Later research showed that Müller cells of the central cone in the foveolar area are biochemically, functionally and morphologically different from both the Z-shaped Müller cells of the perifovea and the straight Müller cells of the peripheral retina [16, 17]. The latter two types are closely intertwined with retinal neuronal cells, whereas MCC cells are not, thus MHs involving only the MCC may indeed occur without PR damage. Earlier, it was also observed by Gass that in some cases macular holes develop after pseudocyst formation in the inner and outer layers of the retina (1A holes) and other cases begin with tractional detachment from the pigment epithelium (PE) of the foveal neurosensory retina (1B holes) [18]. A recent investigation by Chung and Byeon suggested that 1A cystic holes are caused by dehiscence of MCC in the foveola centralis (dehiscent -type A- holes) with few outer foveal tissue defects, while 1B holes develop into tearing (type B) holes, where the foveal wall is torn along the Z-shaped Müller cells and substantial outer foveal tissue loss can occur [19]. They also showed that type B holes had smaller ELM-GCL distance measured on their walls at the largest diameter compared to type A holes. More recently, a review of follow-up OCT images of iFTMH patients confirmed the aforementioned two hole formation mechanisms and identified other mechanisms in iFTMH formation as well: e.g., both degenerative lamellar macular holes associated with epiretinal proliferation (EP) and cases with macular pucker can develop into FTMHs due to tangential tractional forces, whereas subretinal fluid may exert pressure on the fovea from behind resulting in foveal rupture [20]. In addition, the role of cystic cavities of FTMH walls was proposed in hole size enlargement and elevation of the neurosensory retina from retinal pigment epithelium (RPE) [20].

The aim of the present study was to identify preoperative OCT parameters that can predict postoperative BCVA. We put emphasis on parameters that reflect recent understanding on the pathoanatomy of iFTMH formation, i.e. that type B holes associated with potential photoreceptor loss tend to have larger base diameter (B) and smaller ELM-GCL distance (A) as measured on the MH walls [19]. Parameters selected by stepwise multiple logistic regression were used to create the new A/B index (A: smaller ELM-GCL distance as measured on the MH walls at the largest diameter/ B: base diameter (B) Fig. 1).

## Patients and methods

### Patients

This study is a retrospective, non-randomized, interventional consecutive case series of patients who were operated at our institution for idiopathic full thickness macular holes (iFTMH) by two experienced surgeons (AV, LT) between January 2016 and March 2022. The examination process was approved by the Regional Ethical Committee (RKEB approval 5918–2021) and adhered to the tenets of the Helsinki Declaration and informed consent of patients was obtained. Clinical charts of 76 patients (78 eyes) were reviewed. Only cases that were pseudophakic at the last visit, had closed MH, had follow up of at least 12 months and had good quality SD-OCT images available at each visit were included. 32 eyes of 30 patients were analysed (causes of non-inclusion were as follows: high myopia (above -12.0D, 9), previous retinal detachment (12), previous trauma (2), non-closure (6), possible cataract (non pseudophakic) at the last visit (8) and non-suitable (TD-OCT)/low quality OCT images (9)). Baseline and demographic data of patients (gender, age, duration of symptoms, where available) were recorded. Pre- and postoperative BCVA were recorded in logMAR units and Snellen visual acuity, the latter was converted into logMAR units for statistical analysis. OCT images taken at 6 and 12 months (M) visits and at any time afterwards were reviewed. Follow-up periods ranged between 12—83 months.

### Pars plana vitrectomy

All patients were operated on with standard 23 g vitrectomy. During the operation PVD was induced with triamcinolone acetate assistance. Brilliant Blue-G was used to stain ILM which was removed up to the vascular arcades. In the case of large holes (above 400 µm), the inverted ILM flap technique [3] was used. When epiretinal proliferation (EP) was present around the hole, the EP tissue was embedded into the hole as described [21]. In the end of the operation, eyes were filled with C3F8 (11%) or SF6 (20%) gas and the patient was asked to hold face-down position for one week. Phacoemulsification was performed together with the pars plana vitrectomy in cases where cataract was present, or later if cataract developed subsequently.

### OCT analysis

Heidelberg Spectralis OCT (Heidelberg Engineering, Heidelberg Germany) was used to acquire images at all follow-up visits. Scan parameters of the OCT image were as follows: scan angle 20°, size x: 512 pixels (5.7 mm), size y: 496 pixels (1.9 mm), scaling x: 11.07 µm/pixel, scaling y: 3.87 µm/pixel, number of B scans: 25, Pattern size: 20° × 20° (5.7 × 5.7 mm), distance between B scans 236 µm. OCT images of patients at 6 M, 12 M and the last (12-83 M) visit were analysed. All measurements were made at the OCT cross sectional image which was closest to the center of the hole/ largest MH diameter. At each visit, base diameter (B), ellipsoid zone defect, MH diameter (smallest distance between MH walls), MH height, and ELM-GCL distance on both MH walls were measured. The smaller of the two measurements (A) Of ELM-GCL distance was selected for calculation of the A/B index. The following indices were calculated: macular hole index (MHI, maximum height/base diameter), diameter hole index (DHI, minimum hole diameter/maximum hole diameter) tractional hole index (THI, maximum height/minimum hole diameter) MHI, DHI, THI and A/B indices were calculated as illustrated in Fig. 1.

### Statistical analysis

The correlation of all measured preoperative parameters and indices with postoperative BCVA at 6 M, 12 M and at last visit were examined with Pearson correlation. All parameters with significant correlations (*p* ≤ 0.05) were entered into a multiple logistic regression model, with forward stepwise selection employed and BCVA at 6 M, 12 M and final visit as dependent variable. The independence of the entered parameters was checked with Durbin-Watson statistics. The parameters selected as most significant in predicting long term postoperative BCVA were used to create a new index, A/B (smaller ELM-GCL distance measured at the largest hole diameter/base diameter) . Akaike Information Criterion (AIC) was used to estimate parsimony and confirm the best fitted models. Finally, the area under the receiver operating curve of the most significant parameters at each postoperative visit were examined to evaluate the ability of parameters/indices to predict postoperative BCVA of < 0.3 logMAR (> 20/40). Statistical analysis was performed with IBM SPSS Statistics for Windows (version 24.0; IBM Corp., Armonk, NY).

## Results

32 eyes of 30 patients who underwent successful MH surgery were examined, mean age was 70.63 years (95% CI 68.04 to 73.21), and mean duration of symptoms was 11.64 weeks (95% CI 7.89 to 15.39). Mean postoperative follow-up time was 22.22 months (12–83 months), and 81.25% of patients were women.

**Fig. 2:**
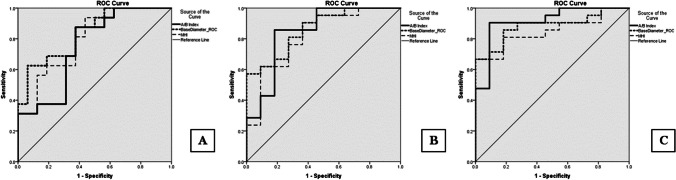
Receiver operating characteristic (ROC) curves for the parameters predicting postoperative Best Corrected Visual Acuity (BCVA) (< 0.3 LogMAR, > 20/40). **A**: BCVA postoperative 6 months; **B**: BCVA postoperative 12 months; **C**: BCVA postoperative last visit. The ROC curve of the A/B Index indicated that it had the strongest predictive value at the postoperative 12 months and last visit

In univariate Pearson correlation analysis, MHI, base diameter (B), ELM-GCL distance (A), smallest diameter, preoperative BCVA showed significant (*p* < 0.05) correlation with BCVA at 6 M, 12 M and last visits (Table 1, Fig. 1). DHI, THI, MH height, age and duration of symptoms did not significantly correlate with any postoperative BCVA (Table 1). There was a significant correlation between A, sum of ELM elevation, and preoperative visual acuity (Table 1). ELM-GCL distance negatively correlated with MH minimal diameter, too (r = -0.426, p = 0.008). To predict postoperative EZ defect, we calculated the difference of base diameter and the sum of elevated ELM on both sides of the FTMH (column 3 in Table 1), similarly to the method reported earlier [8]. This difference showed weak correlation with 12 M postoperative BCVA and borderline values with other postoperative and preoperative BCVAs. However, it did correlate with postoperative EZ defect at 6 M, 12 M and last visit (r = 0.428, 0.442, 0.445, *p* = 0.009, 0.008, 0.009, respectively). Multiple logistic regression showed that base diameter (B) was the best predictor for postoperative 6 M BCVA (R = 0.53, p = 0.002) base diameter (B) and preoperative BCVA for postoperative 12 M BCVA (R = 0.575, *p* = 0,001 and R = 0.69, *p* = 0.008) and base diameter (B) and smaller ELM-GCL distance (A) for the last BCVA (R = 0.568, p = 0.001 and R = 0.662, *p* < 0.001) (Table 2)*.* Since the latter two parameters are important in identifying type A and B iFTMHs, they were used to create a new index, A/B (shortest ELM-GCL distance as measured on MH walls at the largest diameter (A) / base diameter (B) Fig. 1.). AIC was lowest for MHI at 6 M, preoperative BCVA and B at 12 M and B and A at last visit (Supplementary Table 1). Correlation of the A/B index with postoperative BCVA was examined and showed significant correlation at each visit, with increasing correlation coefficient and significance (6 M: r = 0.410 *p* < 0,05,12 M: r = 0.473, *p* < 0,01 last: r = 0.535 *p* < 0.01, Table 1). AU-ROC analysis showed that B, MHI and A/B Index all predicted BCVA ≤ 0.3logMAR relatively well. MHI and B yielded the greatest AUC value at 6 M (0.797, *p* = 0.004 and 0.836, *p* = 0.001 respectively, Fig. 2A and B and Table 3) and the new A/B index at 12 M and at final visit (0.844, *p* = 0.002 and 0.913, *p* = 0.003 respectively, Fig. 2B, C and Table 3). Notably, we found cut-off values (0.43–0.53) for the MHI index similar to the reported value (0.5) [9].

**Table 1:** Results of univariate correlation analysis

	ELM-GCL distance	ELM elevation sum	Base diameter minus ELM elevation sum	MHI	Age	Base Diameter	MH Height	Min Diameter	THI	DHI	A/B Index	preop. BCVA
Preop. BCVA	-0.532**	0.553**	0.082	-0.362*	-0.097	0.397*	0.090	0.409*	-0.179	0.087	0.367*	1
BCVA postop. 6 month	^−0.386*^	0.250	^0.309^	^−0.586**^	0.191	^0.530**^	0.082	^0.422*^	0.011	0.086	^−0.410*^	0.488**
BCVA postop. 12 month	^−0.375*^	0.459**	^0.334*^	^−0.540**^	0.239	^0.575**^	0.151	^0.511**^	-0.026	0.156	^−0.473**^	0.606**
Last BCVA	^−0.535**^	0.427*	^0.254^	^−0.588**^	0.218	^0.568*^	0.100	^0.455**^	0.011	0.104	^−0.535**^	0.520**

The body of the table displays the respective r values. Abbreviations: ELM:external limiting membrane GCL: ganglion cell layer MHI: macular hole index MH: macular hole THI: traction hole index DHI: diameter hole index BCVA: best corrected visual acuity *p < 0.05, **p < 0.01 † Correlation for A/B index was calculated after the completion of multivariate regression

**Table 2:** Multiple logistic regression analysis of factors predicting postoperative BCVA at different time points

Visit	Parameter	R	R^2^	*p*
6 months	Base Diameter	0.530	0.281	0.002
12 months	Base Diameter	0.575	0.331	0.001
	Preoperative BCVA	0.690	0.476	0.008
Last visit	Base Diameter	0.568	0.323	0.001
	ELM-GCL distance	0.662	0.438	0.000

**Table 3:** ROC curve data and cutoff values for postoperative BCVA < 0.3 LogMAR

**6 months**							
Parameter	AU-ROC	95% CI	Cutoff value	Youden’s index	Sensitivity (%)	Specificity (%)	*p*
Base Diameter	0.836	0.699–0.972	749.5	0.563	62.5	93.7	0.001
MHI	0.797	0.643–0.950	0.43	0.5	93.8	56.2	0.004
A/B index	0.75	0.578–0.922	0.168	0.5	87.5	62.5	0.016
**12 months**							
Base Diameter	0.828	0.676–0.980	788.5	0.571	57.1	100	0.001
MHI	0.778	0.608–0.948	0.53	0.528	61.9	90.9	0.004
A/B index	0.844	0.690–0.999	0.168	0.675	85.7	81.8	0.002
**Last visit**							
Base Diameter	0.883	0.766–1.000	835.0	0.667	66.7	100	< 0.001
MHI	0.848	0.716–0.981	0.48	0.628	81	81.8	0.001
A/B index	0.913	0.806–1.000	0.168	0.814	90.5	90.9	0.003

Abbreviations: ROC: receiver operating characteristics AU-ROC: area under the ROC curve CI: confidence interval MHI: macular hole index

## Discussion

In this study, we evaluated the predictive value of several indices and preoperative OCT parameters for short and long term postoperative BCVA, in cases of successfully repaired iFTMHs. Some indices (DHI, THI) that correlated significantly with postoperative visual acuity in earlier works did not show correlation in our cohort. The probable reason for this is that we examined only successfully closed MHs, where closure or non-closure was not affecting postoperative visual acuity. Similarly, a recent study [22], examining a cohort with 98% closure rate did not find significant differences in functional or anatomical outcomes for groups separated based on reported cut-off values of DHI, MHI and THI. With contemporary surgical techniques iFTMH closure rates are above 90% in most studies (in our case 92.3%), and the relevance of former indices has been questioned [22]. In this study, in order to achieve MH closure, various surgical techniques, such as use of SF6 or C3F8 gases as well as inverted ILM technique for holes larger than 400 µm were allowed, according to the surgeons’ preference, similarly to other studies investigating visual outcomes after MH surgery. According to literature data, use of SF6 or C3F8 tamponade does not influence the outcomes or complications of MH surgery [23, 24]. Inverted ILM flap technique improves closure rate in large macular holes, but does not influence postoperative visual acuity in smaller holes [25, 26]. Thus we assume that such variations in surgical technique do not influence the results presented in our study. Once closure is achieved, postoperative BCVA probably depends on MH formation mechanisms and initial PR damage as well as repair processes after closure. Since no visual improvement is observed in non-closed MHs, involving these cases creates a strong statistical bias, only factors predicting MH closure seem to influence final VA. Our goal was to better understand the mechanisms of VA improvement after successful MH closure and find the best predictors of long-term visual improvement, therefore we omitted non-closure cases, similarly to many previous studies [8, 19, 27].

Since postoperative vision of iFTMH patients varies widely, its prediction remains an important goal [28]. Recently, mechanisms of MH formation and repair processes after MH closure were investigated to better understand changes in VA of MH patients. After MH closure, a slow gradual improvement in vision is observed that continues beyond one year, at least until the 3rd postoperative year [29]. This improvement coincides with the regeneration of outer retinal layers, and it is supposed that first ELM, then ONL and finally EZ closure occurs [30]. This regeneration can take place even in the case of very large holes, where inward movement of PR outer segments and outward movement of the inner layers of the fovea was observed, similarly to the organization of foveal structures during retinal development [31]. This “foveation” process can take several years and ultimately even the EZ layer of the large holes can be completely regenerated. The organizing cell layer of foveation is probably the RPE and injury to RPE cells can hinder foveal regeneration, even after successful MH closure [32]. In accordance, high resolution adaptive optics OCT examinations of healing MHs showed inward migration of PRs toward the MH center and restoration of the continuous PR layer in the fovea but with decreased final PR density since lost PR cells cannot be replaced by cell division [33].

In iFTMHs, several factors can lead to PR loss. As FTMHs open, and their walls become oedematous, photoreceptor outer segments are distanced from RPE cells and a slow degeneration process begins [20]. On OCT, bumpiness of the MH edges and RPE granular deposits were proposed signs of PR damage and were shown to be associated with less postoperative BCVA gain [27]. The later the vitrectomy and MH closure happen, the longer the deterioration of PR outer segments continues. Timely operation of FTMHs is therefore important as shown by Steel and co-workers [5], who suggested a cut-off of 4 months after beginning of symptoms as prognostic factor for postoperative BCVA. In our study, patients were uncertain about the beginning of their symptoms in many cases or accidentally perceived visual loss when they closed one eye. According to this, we could not analyse the correlation with duration of symptoms. On the other hand, postoperative BCVA correlation with base diameter (B) was significant at all postoperative visits, larger B possibly indicating more PR outer segment damage in most cases when they were separated from the RPE on a larger area. Moreover, the length of elevated neurosensory retina (sum of elevated ELM segments, Fig. 1, Table 1) also correlated with preoperative and variably with postoperative BCVA, indicating the presence of initial PR damage and, perhaps, variable impairment or repair of damaged outer segments.

Photoreceptor loss can also depend on the type of MH. In type A holes only the MCC is supposed to be lost during hole formation (pseudocyst according to Gass) whereas in type B holes photoreceptor damage may also occur due to more lateral tearing along the Z-shaped Müller cells of the foveal walls (hole with neurosensory retinal detachment according to Gass) [18]. Chung and Byeon, in their study showed that type B holes have smaller ELM-GCC distance as measured on the MH wall, compared to type A holes [19]. These results are corroborated by our findings, showing that ELM-GCL distance correlated with postoperative BCVA at all visits, moreover, correlation coefficients and significance improved with longer term follow-up. Remarkably, the ELM-GCL distance also correlated with preoperative BCVA, suggesting that hole type may indeed indicate initial PR loss (Table 1). Moreover, significant negative correlation was found between ELM-GCL distance and minimal MH diameter (the smaller the ELM-GCL distance, the larger the MH, r = -0.426, p = 0.008) indicating that smaller ELM-GCL distance is also associated with worse MH morphology. Stepwise multiple logistic regression showed that long term BCVA was best predicted by B (base diameter) and A (smaller ELM-GCL distance as measured on the walls of the MH at its largest diameter), which also supports the above hypothesis. Based on these results, we established a new index, A/B. This index proved very efficient in the prediction of long-term visual acuity as shown by AU-ROC results. We assume that smaller ELM-GCL distance results mainly from loss of the outer nuclear layer and to a lesser extent, inner nuclear layer (Fig. 1, coloured panel), that is, loss of photoreceptor cell bodies (cell nuclei) and some bipolar cells. In accordance with this, by immunofluorescence microscopy, Ezra and co-workers found numerous cell nuclei in MH opercula excised during surgery, and in 50% of the examined opercula more than 5 of the nucleated cell bodies proved to be cone PR fragments [13]. When cell bodies involving photoreceptor cell nuclei are lost, any later regeneration of PRs is impossible. When only PR outer segments are damaged because of large base diameter and distanced RPE and PR outer segments, regeneration of the outer segments may be possible to some extent. The trend-like changes both in correlation coefficients and AU-ROC values for the parameters, that are gradually decreasing over time for B and MHI and increasing for A/B index emphasize that short term repair and long-term changes in BCVA may depend on distinct processes (Fig. 2, Table 2 and 3). As mentioned above, repair of damaged outer segments (extent of damage reflected by B and sum of ELM elevation, Table 1) can be a faster process, mostly occurring in the first 12 months. Further repair of the central EZ layer, requiring the rearrangement of central PR cells by the foveation process may require more time, this is why A/B index involving ELM-GCL distance reflecting PR cell nuclei loss may be a stronger predictor of long term (over one year) postoperative VA.

Besides type A and B hole formation, recent OCT observational studies indicated that disruption of atrophic lamellar holes, traction exerted by epiretinal membranes (ERM), and subretinal fluid pressure can also contribute to FTMH formation [20]. In this study, we only examined idiopathic FTMHs, without retinal detachment, thus the latter mechanism probably did not contribute to MH formation in our cases. Atrophic lamellar holes are almost always associated with epiretinal proliferation (EP, LHEP) and we had several cases (3/32, 9.4%) with EP in our series. All these cases were treated with embedment of EP and ILM peeling or inverted EP-ILM flap covering of the MH, according to Takahashi and co-workers [21]. Photoreceptor loss in atrophic lamellar holes may be the consequence of slowly occurring degenerative processes, but the final loss may be reflected by a small ELM-GCC distance, too. In their original article, Chung and Byeon involved only MH cases with no longer than 2 months symptom duration and determined the MH type based on the initial OCT appearance. However, in older cases/stage III and IV holes, and those with associated ERM or EP, determination of type may be difficult. Our A/B index involves the main features that distinguish type A and B holes, thus its predictive power may originate from differentiation of type A and B holes. The significant AU-ROC values of A/B index indicate that it can predict long term BCVA not only in cases when vitreomacular tractional forces play a role in MH formation but also when atrophic processes contribute to the PR damage, as in atrophic lamellar holes, however, this proposition needs further investigation.

In a previous study, very significant correlation was found between postoperative 6 M and 12 M VA and preoperative ELM defect area calculated by measurement and virtual flattening of elevated ELM parts in several diameters of iFTMHs [8]. We calculated a similar value in the largest diameter of MHs in our study (base diameter minus ELM elevation sum in Table 1.), but this value showed only weak correlation with 12 M postoperative BCVA and borderline values with other postoperative and preoperative BCVAs. However, it did correlate with postoperative EZ defect at 6 M, 12 M and last visit. Thus, our findings corroborate the significance of outer layer defect measurements in postoperative BCVA prediction up to 12 M, but also show that preoperative measurements in one single MH diameter have less statistical power than several measurements along the circumference of the MH. Our findings also indicate that postoperative BCVA later than 12 M is less strongly predicted by outer segment damage.

This study has several limitations. First, the number of cases is relatively low. Second, since data were collected retrospectively, OCT measurements did not follow a predetermined protocol. Considering the circular nature of the pathomorphology, radial scans could have shown more consistent details than linear scans, as was demonstrated in previous studies[8]. In addition, in several cases, OCT images at follow-up visits were not taken in the follow-up mode of the device, thus postoperative changes may have been measured at slightly altered diameters. On the other hand, the strength of this study is the robustness of statistical analysis. It is also important that iFTMHs of all stages and types (with or without EP) were involved, thus the application of our results to a broad spectrum of cases may be possible.

## Supplementary Information

Below is the link to the electronic supplementary material.

Supplementary file1 (DOCX 17 KB)

## Data Availability

The datasets created and/or analyzed during the current study are available from the corresponding author upon reasonable request.
