# Supplementary material for: Prediction of long-term visual outcome of idiopathic full-thickness macular hole surgery using optical coherence tomography parameters that estimate potential preoperative photoreceptor damage
Source: Graefes Arch Clin Exp Ophthalmol. 2024 May 8;262(10):3181–9. doi: 10.1007/s00417-024-06500-2 (PMC11458759; doi:10.1007/s00417-024-06500-2)
Supplement: Supplementary file 1 — Supplementary file1 (DOCX 17 KB) [file 417_2024_6500_MOESM1_ESM.docx]

| **Postoperative 6 months** | **Model** | **AIC** |
| --- | --- | --- |
|  | Base Diameter | 65.884 |
|  | Preop. BCVA | 67.332 |
|  | **Preop. BCVA, Base Diameter** | **65.254** |
|  | ELM-GCL distance, Base Diameter | 66.142 |
|  | Base Diameter, MH_Height | 67.037 |
| **Postoperative 12 months** | **Model** | **AIC** |
|  | Base Diameter | 49.393 |
|  | **Preop. BCVA** | **42.015** |
|  | Preop. BCVA, Base Diameter | 42.050 |
|  | Base Diameter, ELM-GCL distance | 49.743 |
|  | Base Diameter, MH_Height | 51.108 |
| **Postoperative last** | **Model** | **AIC** |
|  | Base Diameter | 49.310 |
|  | Preop. BCVA | 46.230 |
|  | Preop. BCVA, Base Diameter | 46.511 |
|  | **ELM-GCL distance, Base Diameter** | **45.078** |
|  | Base Diameter, MH_Height | 50.246 |

Supplementary Table 1. Akaike Information Criterion (AIC) calculation results

Parameters with lowest AIC at each visit are typed in bold. Abbreviations: BCVA: best corrected visual acuity, ELM: external limiting membrane GCL: ganglion cell layer MH: macular hole
